# Treatment Guidelines for Atopic Dermatitis Since the Approval of Dupilumab: A Systematic Review and Quality Appraisal Using AGREE-II

**DOI:** 10.3389/fmed.2022.821871

**Published:** 2022-03-09

**Authors:** Stephanie Ghazal, Zainab Ridha, Kathleen D'Aguanno, David Nassim, Andrea Quaiattini, Elena Netchiporouk, Yves Poulin, Sunil Kalia, Danielle Marcoux, Vincent Piguet, Carolyn Jack

**Affiliations:** ^1^Faculty of Medicine, McGill University, Montreal, QC, Canada; ^2^Faculty of Medicine, Université Laval, Quebec City, QC, Canada; ^3^Schulich Library of Physical Sciences, Life Sciences, and Engineering, McGill University, Montreal, QC, Canada; ^4^Division of Dermatology, McGill University Health Center, Montreal, QC, Canada; ^5^Infectious Diseases and Immunity in Global Health, The Research Institute of the McGill University Health Center, Montreal, QC, Canada; ^6^Centre de Recherche Dermatologique du Québec Métropolitain, Quebec City, QC, Canada; ^7^Department of Dermatology and Skin Science, Vancouver Coastal Health Research Institute, Vancouver, BC, Canada; ^8^Department of Pediatrics, Division of Dermatology, Sainte-Justine University Hospital Center, University of Montreal, Montreal, QC, Canada; ^9^Division of Dermatology, Department of Medicine, University of Toronto, Toronto, ON, Canada; ^10^Division of Dermatology, Women's College Hospital, Toronto, ON, Canada; ^11^Divisions of Dermatology, St. Mary's Hospital, Montreal West Island Integrated University Health and Social Services Centre, Montreal, QC, Canada; ^12^Jewish General Hospital, Montreal West-Central Integrated University Health and Social Services Centre, Montreal, QC, Canada

**Keywords:** dupilumab, treatment guideline, atopic dermatitis, systematic review, quality appraisal, AGREE-II

## Abstract

**Introduction:**

Since its approval for adults with moderate-to-severe atopic dermatitis (AD) in 2017, dupilumab has been incorporated into clinical practice guidelines (CPGs). However, recommendations differ internationally, and the quality assessment of their development is unclear.

**Objective:**

We aimed to systematically review and appraise the quality of CPGs for adult AD reported since 2017 and map the recommendations for dupilumab initiation relative to conventional systemic therapy (CST).

**Materials and Methods:**

A literature search was conducted in June 2020 in MEDLINE, EMBASE, SCOPUS, and CINAHL. Twelve CPGs were retrieved. Methodological quality was assessed using the validated Appraisal of Guidelines for Research & Evaluation II tool (AGREE-II). Recommendations were extracted and compared.

**Results:**

AGREE-II median scores per domain of the CPGs were (%, r = range): scope/purpose, 78% (50–96); stakeholder involvement, 54% (28–85); rigor of development, 39% (21–63); clarity of presentation, 85% (69–100); applicability, 27% (6–51); and editorial independence, 76% (42–100). Neither met the threshold of 70% quality criteria for rigor of development nor the applicability domains. Three CPGs met the criteria for recommendation without modification. CPGs' approach to dupilumab initiation was as follows: second line, preferred over CST and nbUVB (*n* = 1/12 CPG); second line, equivalent to CST or nbUVB (*n* = 3/12 CPGs); third line, after nbUVB or CST (*n* = 5/12 CPGs); and fourth line after nbUVB and CST (*n* = 2/12). No consensus was reached for *n* = 1/12 CPG.

**Conclusion and Relevance:**

Dupilumab is now incorporated into CPGs for adult AD. These CPGs exhibited good quality in scope/purpose, clarity, and editorial independence domains. However, none met AGREE-II criteria for methodological rigor/applicability. Gaps were found in mechanisms for updates, facilitators/barriers, resource implications, and stakeholder involvement. Only *n* = 3/12 CPGs met quality criteria for recommendation without modifications. Of these, two favored a conservative sequential approach for the initiation of dupilumab relative to CST, while one did not reach consensus. Our findings highlight divergent recommendations AD treatment, underlining a need to incorporate quality criteria into future guideline development.

## Introduction

Atopic dermatitis (AD) is the most common chronic inflammatory skin disease worldwide, affecting up to 20% of children (1–5). Prevalence rates in adults can be as high as 10% (6, 7). AD management is typically based on a short-term reactive treatment of acute flares and long-term maintenance therapy (8, 9). In severe or refractory cases, systemic therapy is often warranted (10, 11). While systemic corticosteroids have long been approved by the Food and Drug Administration (FDA), their use, especially long-term, is discouraged due to the breadth of cumulative adverse effects (12). Traditional antimetabolite immuno-modulators, such as azathioprine, mycophenolate mofetil, cyclosporine, and methotrexate, are often used off-label to control severe diseases (8, 9, 13, 14). Dupilumab is the first therapy to be approved for moderate-to-severe AD that does not respond to topical therapies based on large, randomized, double-blind placebo-controlled clinical trials (10, 15–28). More approvals for novel systemic targeted therapies for AD are anticipated in the next few years, including biologics and small molecules such as Janus kinase (JAK) inhibitors (29). Since access to targeted therapies may be restricted by cost, clear guidelines specifying the sequence of available immunomodulating agents in treatment algorithms remain an outstanding need.

The most widely adopted guidelines for AD management were published by the American Academy of Dermatology in 2014; however, these predate the approval of dupilumab, leaving a gap of evidence-based, practical recommendations for up-to-date management of adult AD (8, 9). A number of recent clinical practice guidelines (CPGs) and recommendations from various groups were developed internationally to incorporate dupilumab in treatment algorithms (30–42). To the best of our knowledge, the quality of these CPGs' methods and development processes have not yet been assessed. Furthermore, recommendations vary across CPGs, particularly with regard to indications on how to initiate, sequence, or combine systemic therapies.

To address this gap, we conducted this systematic review of CPGs for adult AD published since the approval of dupilumab in 2017. We aimed to assess the quality of methods and rigor of development processes of CPGs and map their recommendations regarding the position of dupilumab in their treatment algorithms.

## Materials and Methods

### Database

A systematic literature search was conducted on June 3, 2020 in MEDLINE, EMBASE, SCOPUS, and CINAHL. The search was limited to English articles published after 2017 since dupilumab received FDA approval in March 2017, European Medicines Agency (EMA) approval in September 2017, and Health Canada approval in November 2017 (43–46).

### Search Terms

The search terms were decided on *via* consultation with AD experts, as well as methodologists with expertise in systematic reviews and quality appraisals. The following search terms were chosen: “atopic dermatitis” or “eczema” and “dupilumab” or “Dupixent” or “regn 668” or “sar 231893.” The rationale for choosing these terms was based on the reasoning that up-to-date guidelines for AD management in adults should include dupilumab in their treatment algorithm as the first biologic option with AD disease-specific regulatory approval for efficacy and safety.

### Article Selection

**Figure 1:**
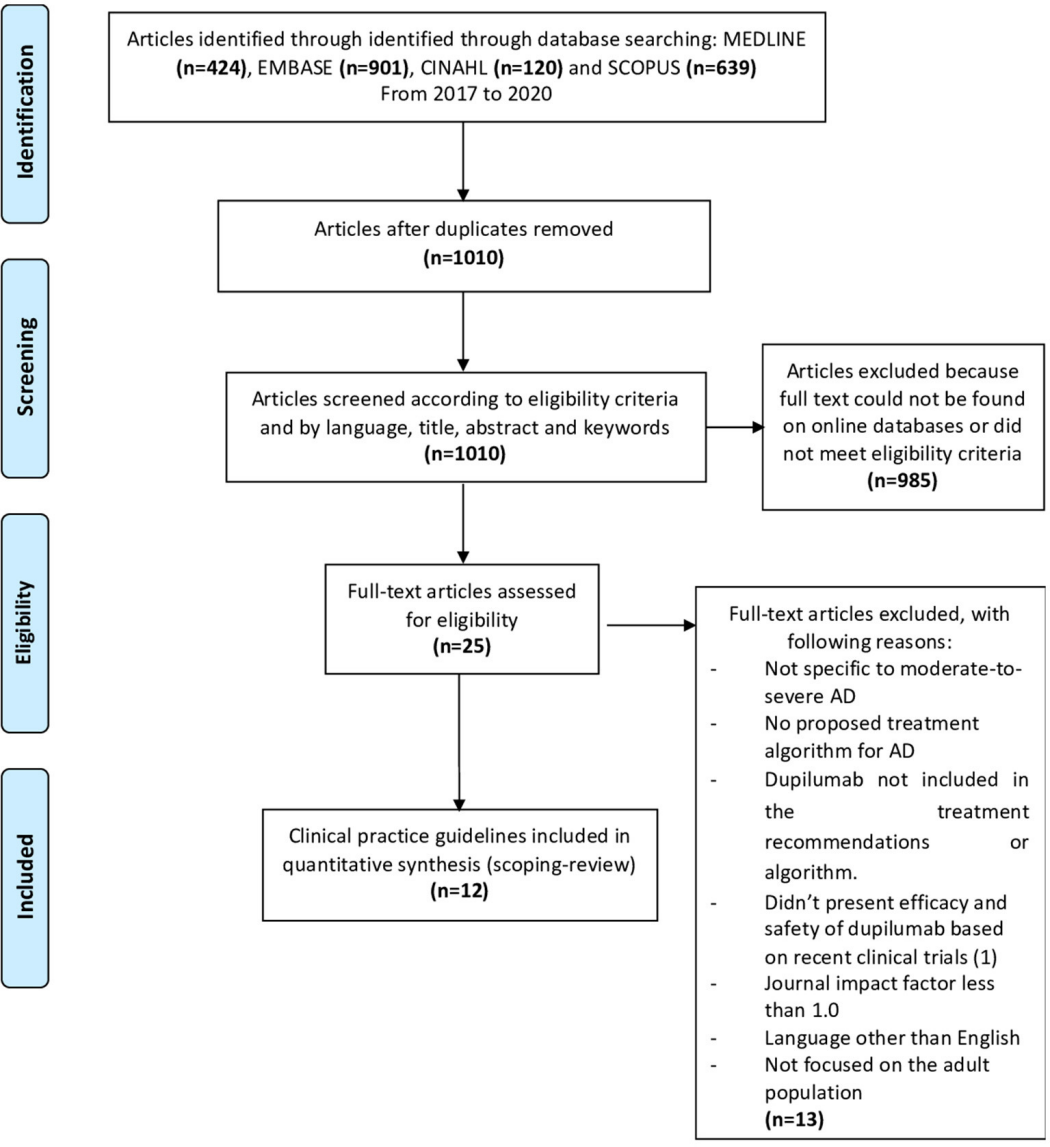
PRISMA flowchart for selection of studies.

Results from MEDLINE, EMBASE, SCOPUS, and CINAHL were combined and exported to Endnote, where duplicates were removed. Two reviewers (SG & ZR) independently screened the articles containing recommendations for dupilumab's initiation in the management of AD by title and abstract when available on the Rayyan software using predetermined exclusion and inclusion criteria (Figure 1) (47).

### Exclusion Criteria

Articles were excluded if they met one or more of the following exclusion criteria:

- Not specific to management of AD.
- Not focused on the adult population.
- Case reports, case series, summaries, or abstracts.

### Inclusion Criteria

The remaining articles were screened based on full content and were included only if they met both of the following inclusion criteria:

- Included treatment recommendations, consensus guidelines, position statements, or treatment algorithms for adults with moderate-to-severe AD.
- Included dupilumab in their treatment recommendations or algorithm.

Discrepancies between the two reviewers were resolved by discussion. If an agreement was not reached, a third reviewer (CJ) resolved the discrepancies.

### Article Characteristics

Two independent reviewers (SG and ZR) extracted the following information for each article included for review: authors, publication date, country of development, patient category described, scoring tool used to assess AD severity, and the method used to reach a consensus based on recommendations.

### Assessment of Guidelines Quality

The quality of CPGs was independently assessed by three reviewers (ZR, KD, and DN) using the validated Appraisal of Guidelines for Research and Evaluation II (AGREE-II) instrument. AGREE-II is an online tool used to evaluate the quality of methods and rigor of development of published CPGs (48–50). It is comprised of 23 items organized into six domains: scope and purpose, stakeholder involvement, rigor of development, clarity of presentation, applicability, and editorial independence (Table 1). Each item is scored on a 7-point scale from 1 (strongly disagree) to 7 (strongly agree). All reviewers completed the AGREE-II training and practice exercise before starting the appraisal.

**Table 1:** Presentation of the 23 criteria evaluated in each of the six AGREE-II quality instrument domains.

**AGREE-II domain**	**Criteria evaluated**
1. Scope and purpose	• The overall objectives of the guideline are specifically described
	• The health questions covered by the guideline are specifically described
	• The population to whom the guideline is meant to apply is specifically described
2. Stakeholder involvement	• The guideline development group includes individuals from all relevant professional groups
	• The views and preferences of the target population have been sought
	• The target users of the guideline are clearly defined
3. Rigor of development	• Systematic methods were used to search for evidence • The criteria for selecting the evidence are clearly described • The strengths and limitations of the body of evidence are clearly described • The methods for formulating the recommendations are clearly described • The health benefits, side effects and risks have been considered in formulating the recommendations • There is an explicit link between the recommendations and the supporting evidence • The guideline has been externally reviewed by experts prior to publication • A procedure for updating the guideline is provided
4. Clarity of presentation	• The recommendations are specific and unambiguous • The different options for management of the condition or health issue are clearly presented • Key recommendations are easily identifiable
5. Applicability	• The guideline describes facilitators and barriers to its application • The guideline provides advice and/or tools on how the recommendations can be put into practice • The potential resource implications of applying the recommendations have been considered • The guideline presents monitoring and/or auditing criteria
6. Editorial independence	• The views of the funding body have not influenced the content of the guideline • Competing interests of guideline development group members have been recorded and addressed

After the reviewers independently scored each CPG, scores were revealed and the domain percentages were calculated following the AGREE-II methodology as follows: (obtained score–minimum possible score)/(maximum possible score–minimum possible), where the “obtained score” is the sum of the appraisers' scores per each item. The AGREE-II instrument does not set a threshold of domain percentage score to differentiate quality. Instead, the manual leaves this cut-off at the discretion of the appraisers. To establish our threshold, a literature review of articles using the AGREE-II tool was performed. Reviews implementing this instrument established an arbitrary threshold of >70% to determine high-quality guidelines (51–53). As such, we used this published threshold to define high quality.

Finally, an overall assessment was attributed to each guideline. Although the AGREE-II instrument does not provide a specific rubric, it recommends that overall CPG quality assessment should be inferred from the domain scores, as well as the independent reviewers' judgment. The overall assessment included an average score of the CPG, and whether the reviewers recommended, recommended with modifications, or did not recommend the CPG.

### Risk of Bias Assessment

As per AGREE-II, the quality of CPGs is defined as “the confidence that the potential biases of guideline development have been addressed adequately and that the recommendations are both internally and externally valid and are feasible for practice.” The use of the AGREE-II tool allows appraisers to evaluate bias in the editorial independence and the rigor of development of published CPGs.

### Mapping of Recommendations

Two reviewers (SG and ZR) extracted each CPG's recommendations regarding the approach to initiating dupilumab in the treatment algorithms. Different approaches were identified, and guidelines were categorized based on the recommended sequence of the initiation of dupilumab. Approaches were categorized as rapid, conservative, and a slow sequential approach, based on the steps recommended prior to the introduction of dupilumab. A rapid sequential approach (1) was defined as initiation of dupilumab as second-line treatment after topicals. This classification was further subdivided as 1A: dupilumab is equivalent to antimetabolite/conventional systemic therapies, 1B: dupilumab is preferred over antimetabolite/conventional systemic therapies or narrow-band UVB phototherapy (nbUVB), and 1C: dupilumab is an equivalent choice to nbUVB. A conservative sequential approach (2) places dupilumab as the second-line treatment after the failure of topicals as well as an alternative, second-line therapeutic modality; in 2A, nbUVB is second and in 2B, antimetabolite/conventional systemic therapies are second. Finally, a slow sequential approach places dupilumab as 4th line, after the failure of topicals and 2nd line (nbUVB) and 3rd line modalities (antimetabolite/conventional systemic therapies).

## Results

### Guideline Selection and Characteristics

The search yielded 424 articles on MEDLINE, 901 on EMBASE, 120 on CINAHL, and 639 on SCOPUS, with a total of 1,010 articles to be screened after removing duplicates (Figure 1). After abstract screening, 985 articles were excluded, with the remaining 25 articles assessed in full text. A total of 12 CPGs were retrieved (Figure 1; Table 2).

**Table 2:** Characteristics of the included clinical practice guidelines selected for review.

**References**	**Country**	**Severity of AD**	**Scoring tools used to assess AD severity**		**Consensus method**
			**Objective tools (CRO)**	**Subjective tools (PRO)**	
Ariens et al. (30)	European countries	Severe	n/s^a^		n/s
Boguniewicz et al. (31)	USA	Moderate–Severe	BSA>10	HRQoL^*^ (sleep loss, work productivity, social life)	Delphi
			Individual lesions with moderate-severe features		
Boguniewicz et al. (32)	USA	Moderate–Severe	n/s		n/s
Brar et al. (33)	USA	Severe	BSA >10	HRQoL	n/s
			Individual lesions with severe features		
Calzavara et al. (34)	Italy	Moderate–Severe	EASI^b^	DLQI >10	Delphi
				HRQoL^*^(Sleep loss) NRS >7	
Damiani et al. (35)	Italy	Severe	EASI 23–72	n/a	Committee
			SCORAD > 50		
Lopes et al. (36)	Portugal	Severe	SCORAD > 50	DLQI > 10	n/s
Lynde et al. (37)	Canada	Moderate–Severe	BSA > 10	DLQI > 10	Committee
			PGA > 3	NRS > 4	
Nowicki et al. (42)	Poland	Moderate–Severe	n/s		n/s
Smith et al. (38)	Australia	Moderate–Severe	BSA > 10	DLQI > 10	Delphi
			PGA > 3	NRS > 4	
				Failure of topical treatment	
Thyssen et al. (39)	Nordic countries	Moderate–Severe	EASI^*^	DLQI^*^	Delphi
			SCORAD^*^	POEM^c^^*^	
Wollenberg et al. (40, 41)	European countries	Moderate–Severe	SCORAD 25–50	n/a	Committee

a *EASI and SCORAD were used as monitoring tools for treatment effect*.

b *Moderate-severe AD was defined as EASI > 16 or EASI < 16 with at least 1 of the 4 following conditions: localized to face, hands, genitals, itch with NRS < 7, DLQI > 10, sleep disturbance with NRS > 7*.

c *Unique in that it combines 2 subjective questions (itch and impact on sleep)*.

* *Did not specify numerical scores*.

*BSA, body surface area; Committee, a committee or panel of experts discussed specific topics, reviewed the literature, and created recommendations based on expertise; CRO, clinician-reported outcome; Delphi, a Delphi or modified Delphi approach was used to reach consensus. DLQI, Dermatology Life Quality Index; EASI, Eczema Area and Severity Index; HRQoL, Health Related Quality of Life; n/a, not applicable; n/s, not specified; NRS, pruritus Numerical Rating Scale; PGA, Patient Global Assessment; POEM, Patient-Oriented Eczema Measure; PRO, patient-reported outcome; SCORAD: SCORing Atopic Dermatitis*.

### AGREE-II Scores

#### Scope and Purpose

The median score for scope and purpose domain items, including specific description of the CPG objectives, health question, and target population, was 78% (range 50–96%). Damiani et al. (35) and Nowicki et al. (42) did not meet the 70% threshold due to gaps in describing their target population.

#### Stakeholder Involvement

The median score for stakeholder involvement items (diverse stakeholders involved, patient perspectives sought, target guideline users defined) was 54% (range 28–85%). The only CPGs to obtain AGREE-II scores above 70% were Smith et al. (38) and Wollenberg et al. (40). The patients' point of view and preferences were only taken into account in the Wollenberg et al. (40) and Lopes et al. (36) CPGs.

#### Rigor of Development

In this domain, the Agree-II instrument items are extensive and detailed. They include the use of external experts' review, the use of systematic methods, description of criteria used for evidence selection, disclosure of strengths and limitations, documentation of methods for formulating recommendations, and reference to explicit links to guidance with supporting evidence. Additional items include considerations of health benefits, side effects and risks, and a procedure for updating CPGs. No guideline met the above criteria with a score >70%. The median score of CPGs was 39% (range 21–63%). While most guidelines included health benefits, side effects, and risks in their recommendations, only two guidelines (35, 40) provided a procedure for updating their recommendations. Calzavara et al. (34), Smith et al. (38), and Wollenberg et al. (40) had their guidelines externally reviewed. Thyssen et al. (39) was the only guideline to adequately describe systematic methods and criteria used to select evidence.

#### Clarity of Presentation

Unambiguous and specific recommendations, clear management options, and easily identifiable key recommendations are the three criteria included here; this was the highest-scoring domain with a median of 85% (range 69–100%). Nearly all CPGs scored >70%. The guidelines accurately outlined the different treatments for AD, with key recommendations illustrated by flow charts and algorithms.

#### Applicability

The criteria in this domain focus on tools, facilitators, and barriers to the implementation of CPGs, as well as health resource implications and monitoring/auditing criteria. These criteria were the least well met in the CPGs reviewed, as reflected by a median score of 27% in this domain (range 6–51%). No guidelines were scored >70%. Implementation strategies that included tools or recommendations on how to carry out the guidelines in practice were missing. Only Ariens et al. (18) and Thyssen et al. (39) acknowledged the cost/resource implications of their recommendations.

#### Editorial Independence

The median score for editorial independence (independence from funding body or conflicts of interest) was 76% (range 42–100%). Most CPGs clearly stated and addressed the conflicts of interest of their group members; however, the influence of funding bodies on CPG development was not always clarified.

#### Overall Assessment in Considering a Guideline for Recommendation

The CPGs that were reviewed generally performed well (Table 3). However, few CPGs met AGREE-II criteria for stakeholder involvement in particular, and the majority of items required for top AGREE-II quality scoring in the rigor of development and applicability domains were missing. Based on the domain scores and on the three appraisers' personal judgement, three CPGs were recommended without changes, and nine were recommended with modifications.

**Table 3:** Standardized scores for each domain using the AGREE-II instrument.

**References**	**AGREE domaine scores (%)**						**Overall quality (/7)**	**Overall AGREE-II**
	**Scope and purpose**	**Stakeholder involvement**	**Rigor of development**	**Clarity of presentation**	**Applicability**	**Editorial independence**		
Ariens et al. (18)	74	43	26	80	51	92	4	Recommended with modifications
Boguniewicz et al. (31)	83	67	43	85	14	86	4.6	Recommended with modifications
Boguniewicz et al. (32)	85	50	36	89	22	67	3.3	Recommended with modifications
Brar et al. (33)	83	44	21	96	26	81	3.3	Recommended with modifications
Calzavara et al. (34)	91	63	37	69	18	42	2.6	Recommended with modifications
Damiani et al. (35)	50	37	47	74	6	47	3.6	Recommended with modifications
Lopes et al. (36)	74	50	26	91	40	97	3.3	Recommended with modifications
Lynde et al. (37)	74	46	31	78	19	72	3.3	Recommended with modifications
Nowicki et al. (42)	52	28	24	78	26	53	4	Recommended with modifications
Smith et al. (38)	93	72	56	91	36	100	5.6	Recommended
Thyssen et al. (39)	83	65	63	85	39	86	5.6	Recommended
Wollenberg et al. (40)	96	85	55	100	35	94	5.6	Recommended

*Scores presented in this table are the means calculated from the scores of the three independent appraisers. Domain scores were rounded*.

### Mapping CPGs' Recommendations

The approaches of CPGs to the sequence of initiation of dupilumab in the treatment of adult AD were highly variable (Table 4). No single approach appeared in more than three guidelines.

**Table 4:** Recommended time for initiation of dupilumab relative to other treatment modalities, after 1st-line measures and topicals.

**Guideline**	**Type of approach**						
	**Rapid** **sequential**			**Conservative** **sequential**		**Slow sequential**	**No consensus**
	**2A**	**2B**	**2C**	**3A**	**3B**	**4A**	
Ariens et al. (18)						x	
Boguniewicz et al. (31)		x					
Boguniewicz et al. (32)	x						
Brar et al. (33)			x				
Calzavara et al. (34)						x	
Damiani et al. (35)					x		
Lopes et al. (36)					x		
Lynde et al. (37)				x			
Nowicki et al. (42)	x						
Smith et al. (38)				x			
Thyssen et al. (39)							x
Wollenberg et al. (40)					x		

*2. Rapid sequential approach: Dupilumab 2nd (after topicals)*.

*2A: As an equivalent to antimetabolite/conventional systemic therapies*.

*2B: preferred over antimetabolite/conventional systemic therapies or phototherapy*.

*2C: As an equivalent choice to phototherapy*.

*3. Conservative sequential approach: Dupilumab as 3rd line, (after topicals + 2nd intervention)*.

*3A: 2nd = narrow band UVB (nbUVB) phototherapy*.

*3B: 2nd = antimetabolite/conventional systemic therapies*.

*4. Slow sequential approach: dupilumab as 4th line [after topicals+ 2nd (nbUVB) + 3rd (conventional systemic therapy)]*.

*4A: 2nd = nbUVB, 3rd = conventional antimetabolite/conventional systemic therapies or vice versa or one conventional to another prior to dupilumab*.

## Discussion

In this systematic review of international CPGs for adult AD since the approval of dupilumab, we applied the validated AGREE-II instrument to measure and compare methodological quality before addressing recommendations for the use of this targeted on-label therapeutic. We found 12 relevant publications for supporting clinical decisions in the adult AD population; however, according to the validated AGREE-II instrument and a preset 70% threshold for item completion, only three CPGs were recommendable without modifications (38–40). Interestingly, recommendations regarding dupilumab initiation relative to conventional systemic therapy (CST) were highly variable, demonstrating a lack of consensus.

Our analysis of quality domains as per the AGREE-II found that most international guidelines demonstrated high scores in the quality domains of scope and purpose, clarity, and editorial independence. In contrast, AGREE-II criteria were frequently missing in other domains; for example, stakeholder involvement in CPGs development was low and applicability criteria were often unmet. Increasingly, the views of the guidelines' target patient populations are valued, and as such, addressing the patient perspective and incorporating stakeholders into future recommendations will be of high importance. In addition, to meet AGREE-II targets for rigor of development, future guidelines may consider describing in detail the strengths and limitations of the evidence used and/or linking the supporting evidence to their recommendations. Importantly, facilitators and barriers to guideline application in clinical practice must be explicitly addressed for guidelines to meet the AGREE-II criteria. With an exponential rate and volume of translational research evidence, flexible and versatile mechanisms for addressing updates to recommendations will also be crucial to incorporate in future CPGs. Moreover, stakeholder engagement to discuss and define the relative weight of various quality domains in the development of CPGs may be useful.

In this review, a variety of approaches were identified regarding the place of dupilumab initiation in the treatment algorithm for adult AD. These approaches were categorized as rapid, conservative, or slow-sequential, depending on the position of dupilumab as 2nd, 3rd, or 4th line after general measures and topical therapies. Nearly, one-third of the CPGs recommend a rapid sequential approach, introducing dupilumab after topical therapy failure, with two of four CPGs considering this biologic equivalent to antimetabolite/conventional immunomodulators. A more conservative sequential approach was suggested by less than half of CPGs, placing dupilumab as 3rd line after nbUVB or after antimetabolites/conventional systemic therapies. A slow sequential approach was proposed by two CPGs who recommend dupilumab as the 4th line, following the use of phototherapy and conventional systemics. Interestingly, the three CPGs with the highest metrics for quality and recommendable without modification based on the AGREE-II instrument (38–40) also had divergent management approaches, although two of three suggested initiating the anti-IL-4R alpha monoclonal therapy as third-line, after NB-UVB or CST failure. Notably, Thyssen et al. did not reach a consensus with respect to the time of initiation of dupilumab. Given dupilumab's known efficacy and safety, these results may reflect disease heterogeneity, variability in payer or regulatory landscapes, and physician preference and comfort. However, there is a clear need for real-world evidence and comparative studies to address the lack of consensus, in particular now that a march of newer therapies lies ahead. Our review found a crucial element omitted by the majority of CPGs pertained to limitations of access and cost-benefit implications. Although currently approved and available in over 60 countries, pharmacoeconomic barriers and the need for regulatory approval across nations may contribute to the observed discrepancies and heterogeneity in management approaches (44, 54, 55). The variability in the accessibility of phototherapy across nations is another factor that contributes to discrepancies observed across CPGs.

Lastly, in most CPGs, the definition of treatment failure in AD is either too broad or entirely absent. Ariens et al. define treatment failure as discontinuation of the agent due to side effects or ineffectiveness using an adequate dose; however, definitions such as this were not found in other CPGs. Thus, the lack of criteria to define non-response poses challenges in deciding to change management approaches. A standard definition of treatment failure in AD is an important area for future research (40).

## Limitations

A limitation of this study is the fact that the search was conducted on general databases (MEDLINE, EMBASE, SCOPUS, and CINAHL) and did not include a search of systematic review registries (e.g., PROSPERO, the Joanna Briggs Institute database of systematic reviews) or the grey literature (e.g., government and organization websites). However, a search of “atopic dermatitis management guidelines” was performed on Google and did not yield additional results that were not included in our search. Another limitation pertains to the application of the AGREE-II instrument. The AGREE-II instruction manual does not set a threshold to differentiate a high-quality and low-quality CPG. For this reason, it is up to the appraisers to subjectively decide on an acceptable threshold. A threshold of 70% of the items was selected for this review based on evidence precedent, as publications using AGREE-II instrument established this preset point. The overall quality and decision to classify CPGs as “recommendable,” “recommendable with modifications,” or “non-recommendable” is based in part on reviewers' judgement, making this a relatively subjective assessment, and the recommendations are made within the lens of the quality instrument itself. Furthermore, the AGREE II tool does not provide its users with the relative importance for each of the 6 domains. Thus, the scores of an AGREE-II evaluation should be interpreted cautiously, and all existing algorithms and guidelines found in this review contribute meaningful and significant recommendations as aides to clinical practice. Certain AGREE-II items, such as a mechanism for keeping guidelines up-to-date, consideration of potential resource implications of applying the recommendations, or monitoring and/or auditing criteria, may be beyond the scope or budgetary limitations of many existing groups developing such guidelines and may or may not be considered relevant to many practicing dermatologists or clinicians referencing them.

## Conclusion

Our findings highlight a need to consider quality domains and the items used to create criteria for assessment by tools, such as the AGREE-II, into the new generation of evidence-based treatment guidelines for adult AD. Key features to incorporate in future CPGs according to AGREE include diverse stakeholder involvement, mechanisms for guideline implementation in practice, as well as features for adaptation to particular populations and age groups. This will become increasingly important in future AD CPGs given the wide range of options for additional systemic treatments soon to be available.

## Author Contributions

SG and ZR performed the literature search, wrote, and reviewed the manuscript. ZR, KD'A, and DN assessed the quality of the CPGs using AGREE II instrument, wrote, and reviewed the manuscript. AQ, EN, YP, SK, DM, and VP wrote and reviewed the manuscript. CJ reviewed the manuscript and supervised the research activities. All authors contributed to the article and approved the submitted version.

## Conflict of Interest

CJ reports grants from Innovaderm Research, McGill University Department of Medicine, MITACS, Canadian Dermatology Foundation, and Eczema Society of Canada, as well as grants, involvement in clinical studies, and/or consultancy work for Sanofi, Eli Lilly, AbbVie, Novartis, Bausch, Pfizer, Amgen, Celgene, Janssen, Boehringer Ingelheim, Asana, Leo, and UCB. The remaining authors declare that the research was conducted in the absence of any commercial or financial relationships that could be construed as a potential conflict of interest.

## Publisher's Note

All claims expressed in this article are solely those of the authors and do not necessarily represent those of their affiliated organizations, or those of the publisher, the editors and the reviewers. Any product that may be evaluated in this article, or claim that may be made by its manufacturer, is not guaranteed or endorsed by the publisher.

## References

[1] Leung DY Guttman-Yassky E. Deciphering the complexities of atopic dermatitis: shifting paradigms in treatment approaches. J Allergy Clin Immunol. (2014) 134:769–79. 10.1016/j.jaci.2014.08.008 25282559 PMC4186710

[2] Shaw TE Currie GP Koudelka CW Simpson EL. Eczema prevalence in the United States: data from the 2003 National Survey of Children's Health. J Invest Dermatol. (2011) 131:67–73. 10.1038/jid.2010.251 20739951 PMC3130508

[3] Schmitt J Langan S Deckert S Svensson A von Kobyletzki L Thomas K . Assessment of clinical signs of atopic dermatitis: a systematic review and recommendation. J Allergy Clin Immunol. (2013) 132:1337–47. 10.1016/j.jaci.2013.07.008 24035157

[4] Odhiambo JA Williams HC Clayton TO Robertson CF Asher MI. Global variations in prevalence of eczema symptoms in children from ISAAC Phase Three. J Allergy Clin Immunol. (2009) 124:1251–58.e1223. 10.1016/j.jaci.2009.10.009 20004783

[5] Silverberg JI Hanifin JM. Adult eczema prevalence and associations with asthma and other health and demographic factors: a US population-based study. J Allergy Clin Immunol. (2013) 132:1132–8. 10.1016/j.jaci.2013.08.031 24094544

[6] Blaabjerg MS Andersen KE Bindslev-Jensen C Mortz CG. Decrease in the rate of sensitization and clinical allergy to natural rubber latex. Contact Dermatitis. (2015) 73:21–8. 10.1111/cod.12386 25817831

[7] Weidinger S Beck LA Bieber T Kabashima K Irvine AD. Atopic dermatitis. Nat Rev Dis Prim. (2018) 4:1. 10.1038/s41572-018-0001-z 29930242

[8] Eichenfield LF Tom WL Chamlin SL Feldman SR Hanifin JM Simpson EL . Guidelines of care for the management of atopic dermatitis: section 1. Diagnosis and assessment of atopic dermatitis. J Am Acad Dermatol. (2014) 70:338–51. 10.1016/j.jaad.2013.10.010 24290431 PMC4410183

[9] Eichenfield LF Tom WL Berger TG Krol A Paller AS Schwarzenberger K . Guidelines of care for the management of atopic dermatitis: Section 2. Management and treatment of atopic dermatitis with topical therapies. J Am Acad Dermatol. (2014) 71:116–32. 10.1016/j.jaad.2014.03.023 24813302 PMC4326095

[10] Simpson EL Bruin-Weller M Flohr C Ardern-Jones MR Barbarot S Deleuran M . When does atopic dermatitis warrant systemic therapy? Recommendations from an expert panel of the International Eczema Council. J Am Acad Dermatol. (2017) 77:623–33. 10.1016/j.jaad.2017.06.042 28803668

[11] Feldman SR Cox LS Strowd LC Gerber RA Faulkner S Sierka D . The challenge of managing atopic dermatitis in the United States. Am Health Drug Benefits. (2019) 12:83–93. 31057694 PMC6485648

[12] Drucker AM Eyerich K de Bruin-Weller MS Thyssen JP Spuls PI Irvine AD . Use of systemic corticosteroids for atopic dermatitis: International Eczema Council consensus statement. Br J Dermatol. (2018) 178:768–75. 10.1111/bjd.15928 28865094 PMC5901393

[13] Sidbury R Davis DM Cohen DE Cordoro KM Berger TG Bergman JN . Guidelines of care for the management of atopic dermatitis: section 3. Management and treatment with phototherapy and systemic agents. J Am Acad Dermatol. (2014) 71:327–49. 10.1016/j.jaad.2014.03.030 24813298 PMC4410179

[14] Sidbury R Tom WL Bergman JN Cooper KD Silverman RA Berger TG . Guidelines of care for the management of atopic dermatitis: Section 4. Prevention of disease flares and use of adjunctive therapies and approaches. J Am Acad Dermatol. (2014) 71:1218–33. 10.1016/j.jaad.2014.08.038 25264237 PMC4430554

[15] Tsianakas A Luger TA Radin A. Dupilumab treatment improves quality of life in adult patients with moderate-to-severe atopic dermatitis: results from a randomized, placebo-controlled clinical trial. Br J Dermatol. (2018) 178:406–14. 10.1111/bjd.15905 28845523

[16] Gooderham MJ Hong HC Eshtiaghi P Papp KA. Dupilumab: a review of its use in the treatment of atopic dermatitis. J Am Acad Dermatol. (2018) 78(3 Suppl 1):S28-s36. 10.1016/j.jaad.2017.12.022 29471919

[17] Blauvelt A de Bruin-Weller M Gooderham M Cather JC Weisman J Pariser D . Long-term management of moderate-to-severe atopic dermatitis with dupilumab and concomitant topical corticosteroids (LIBERTY AD CHRONOS): a 1-year, randomised, double-blinded, placebo-controlled, phase 3 trial. Lancet. (2017) 389:2287–303. 10.1016/S0140-6736(17)31191-1 28478972

[18] Ariens LFM van der Schaft J Bakker DS Balak D Romeijn MLE Kouwenhoven T . Dupilumab is very effective in a large cohort of difficult-to-treat adult atopic dermatitis patients: First clinical and biomarker results from the BioDay registry. Allergy. (2020) 75:116–26. 10.1111/all.14080 31593343

[19] Deleuran M Thaçi D Beck LA de Bruin-Weller M Blauvelt A Forman S . Dupilumab shows long-term safety and efficacy in patients with moderate to severe atopic dermatitis enrolled in a phase 3 open-label extension study. J Am Acad Dermatol. (2020) 82:377–88. 10.1016/j.jaad.2019.07.074 31374300

[20] Simpson EL Paller AS Siegfried EC Boguniewicz M Sher L Gooderham MJ . Efficacy and safety of dupilumab in adolescents with uncontrolled moderate to severe atopic dermatitis: a phase 3 randomized clinical trial. JAMA Dermatol. (2020) 156:44–56. 10.1001/jamadermatol.2019.3336 31693077 PMC6865265

[21] Cork MJ Thaci D Eichenfield LF Arkwright PD Hultsch T Davis JD . Dupilumab in adolescents with uncontrolled moderate-to-severe atopic dermatitis: results from a phase IIa open-label trial and subsequent phase III open-label extension. Br J Dermatol. (2020) 182:85–96. 10.1111/bjd.18476 31595499 PMC6972638

[22] Paller AS Siegfried EC Thaci D Wollenberg A Cork MJ Arkwright PD . Efficacy and safety of dupilumab with concomitant topical corticosteroids in children 6 to 11 years old with severe atopic dermatitis: a randomized, double-blinded, placebo-controlled phase 3 trial. J Am Acad Dermatol. (2020) 83:1282–93. 10.1016/j.jaad.2020.06.054 32574587

[23] Barbarot S Wollenberg A Silverberg JI Deleuran M Pellacani G Armario-Hita JC . Dupilumab provides rapid and sustained improvement in SCORAD outcomes in adults with moderate-to-severe atopic dermatitis: combined results of four randomized phase 3 trials. J Dermatolog Treat. (2020) 2020:1–12. 10.1080/09546634.2020.1750550 32347763

[24] Silverberg JI Simpson EL Ardeleanu M Thaci D Barbarot S Bagel J . Dupilumab provides important clinical benefits to patients with atopic dermatitis who do not achieve clear or almost clear skin according to the Investigator's Global Assessment: a pooled analysis of data from two phase III trials. Br J Dermatol. (2019) 181:80–7. 10.1111/bjd.17791 30791102 PMC6849829

[25] Simpson EL Gadkari A Worm M Soong W Blauvelt A Eckert L . Dupilumab therapy provides clinically meaningful improvement in patient-reported outcomes (PROs): A phase IIb, randomized, placebo-controlled, clinical trial in adult patients with moderate to severe atopic dermatitis (AD). J Am Acad Dermatol. (2016) 75:506–15. 10.1016/j.jaad.2016.04.054 27268421

[26] Simpson EL Bieber T Guttman-Yassky E Beck LA Blauvelt A Cork MJ . Two phase 3 trials of dupilumab versus placebo in atopic dermatitis. N Engl J Med. (2016) 375:2335–48. 10.1056/NEJMoa1610020 27690741

[27] Simpson EL Bieber T Eckert L Wu R Ardeleanu M Graham NMH . Patient burden of moderate to severe atopic dermatitis (AD): insights from a phase 2b clinical trial of dupilumab in adults. J Am Acad Dermatol. (2016) 74:491–8. 10.1016/j.jaad.2015.10.043 26777100

[28] Sawangjit R Dilokthornsakul P Lloyd-Lavery A Lai NM Dellavalle R Chaiyakunapruk N. Systemic treatments for eczema: a network meta-analysis. Cochrane Database Syst Rev. (2020) 9:CD013206. 10.1002/14651858.CD013206.pub2 32927498 PMC8128359

[29] Drucker AM Ellis AG Bohdanowicz M Mashayekhi S Yiu ZZN Rochwerg B . Systemic immunomodulatory treatments for patients with atopic dermatitis: a systematic review and network meta-analysis. JAMA Dermatol. (2020) 156:659–67. 10.1001/jamadermatol.2020.0796 32320001 PMC7177646

[30] Ariëns LFM Bakker DS van der Schaft J Garritsen FM Thijs JL de Bruin-Weller MS. Dupilumab in atopic dermatitis: rationale, latest evidence and place in therapy. Ther Adv Chronic Dis. (2018) 9:159–70. 10.1177/2040622318773686 30181845 PMC6116085

[31] Boguniewicz M Alexis AF Beck LA Block J Eichenfield LF Fonacier L . Expert perspectives on management of moderate-to-severe atopic dermatitis: a multidisciplinary consensus addressing current and emerging therapies. J Allergy Clin Immunol Pract. (2017) 5:1519–31. 10.1016/j.jaip.2017.08.005 28970084

[32] Boguniewicz M Fonacier L Guttman-Yassky E Ong PY Silverberg J Farrar JR. Atopic dermatitis yardstick: Practical recommendations for an evolving therapeutic landscape. Ann Allergy Asthma Immunol. (2018) 120:10–22.e12. 10.1016/j.anai.2017.10.039 29273118

[33] Brar KK Nicol NH Boguniewicz M. Strategies for successful management of severe atopic dermatitis. J Allergy Clin Immunol Pract. (2019) 7:1–16. 10.1016/j.jaip.2018.10.021 30598172

[34] Calzavara Pinton P Cristaudo A Foti C Canonica GW Balato N Costanzo A . Diagnosis and management of moderate to severe adult atopic dermatitis: a Consensus by the Italian Society of Dermatology and Venereology (SIDeMaST), the Italian Association of Hospital Dermatologists (ADOI), the Italian Society of Allergy, Asthma and Clinical Immunology (SIAAIC), and the Italian Society of Allergological, Environmental and Occupational Dermatology (SIDAPA). G Ital Dermatol Venereol. (2018) 153:133–45. 10.23736/S0392-0488.17.05892-8 29237258

[35] Damiani G Calzavara-Pinton P Stingeni L Hansel K Cusano F Pigatto PDM. Italian guidelines for therapy of atopic dermatitis-adapted from consensus-based European guidelines for treatment of atopic eczema (atopic dermatitis). Dermatol Ther. (2019) 32:e13121. 10.1111/dth.13121 31625221

[36] Lopes A Sokolova A Abreu C Lopes C. Atopic dermatitis host and environment model: revisiting therapeutic options. Eur Ann Allergy Clin Immunol. (2020) 52:4–14. 10.23822/EurAnnACI.1764-1489.125 31789489

[37] Lynde CW Bourcier M Gooderham M Guenther L Hong CH Papp KA . A treatment algorithm for moderate to severe atopic dermatitis in adults. J Cutan Med Surg. (2018) 22:78–83. 10.1177/1203475417733460 29082775

[38] Smith S Baker C Gebauer K Rubel D Frankum B Soyer HP . Atopic dermatitis in adults: an Australian management consensus. Australas J Dermatol. (2020) 61:23–32. 10.1111/ajd.13124 31372984

[39] Thyssen JP Berents T Bradley M Deleuran M Grimstad O Korhonen L . Clinical management of atopic dermatitis in adults: mapping of expert opinion in 4 nordic countries using a modified delphi process. Acta Derm Venereol. (2020) 100:adv00015. 10.2340/00015555-3369 31709450 PMC9128918

[40] Wollenberg A Barbarot S Bieber T Christen-Zaech S Deleuran M Fink-Wagner A . Consensus-based European guidelines for treatment of atopic eczema (atopic dermatitis) in adults and children: part I. J Eur Acad Dermatol Venereol. (2018) 32:657–82. 10.1111/jdv.14891 29676534

[41] Wollenberg A Barbarot S Bieber T Christen-Zaech S Deleuran M Fink-Wagner A . Consensus-based European guidelines for treatment of atopic eczema (atopic dermatitis) in adults and children: part II. J Eur Acad Dermatol Venereol. (2018) 32:850–78. 10.1111/jdv.14888 29878606

[42] Nowicki RJ Trzeciak M Kaczmarski M Wilkowska A Czarnecka-Operacz M Kowalewski C . Atopic dermatitis. Interdisciplinary diagnostic and therapeutic recommendations of the Polish Dermatological Society, Polish Society of Allergology, Polish Pediatric Society and Polish Society of Family Medicine Part II Systemic treatment and new therapeutic methods. Postepy Dermatol Alergol. (2020) 37:129–34. 10.5114/ada.2020.94829 32489345 PMC7262801

[43] Seegraber M Srour J Walter A Knop M Wollenberg A. Dupilumab for treatment of atopic dermatitis. Expert Rev Clin Pharmacol. (2018) 11:467–74. 10.1080/17512433.2018.1449642 29557246

[44] FDA approves new eczema drug dupixent. (2017). Available online at: https://www.fda.gov/news-events/press-announcements/fda-approves-new-eczema-drug-dupixent (accessed March 11, 2021).

[45] Dupixent - European Medicines Agency. (2017). Available online at: https://www.ema.europa.eu/en/medicines/human/EPAR/dupixent (accessed March 12, 2021).

[46] DUPIXENT^®^ (dupilumab injection) now approved by Health Canada for patients with severe asthma - Nov 17 2020. (2020). Available online at: http://sanoficanada.mediaroom.com/2020-11-17-DUPIXENT-R-dupilumab-injection-now-approved-by-Health-Canada-for-patients-with-severe-asthma (accessed March 12, 2021).

[47] Ouzzani M Hammady H Fedorowicz Z Elmagarmid A. Rayyan—a web and mobile app for systematic reviews. Syst Rev. (2016) 5:210. 10.1186/s13643-016-0384-4 27919275 PMC5139140

[48] Dans AL Dans LF. Appraising a tool for guideline appraisal (the AGREE II instrument). J Clin Epidemiol. (2010) 63:1281–2. 10.1016/j.jclinepi.2010.06.005 20605571

[49] Brouwers MC Kerkvliet K Spithoff K Consortium ANS. The AGREE Reporting Checklist: a tool to improve reporting of clinical practice guidelines. BMJ. (2016) 352:i1152. 10.1136/bmj.i1152 26957104 PMC5118873

[50] Brouwers MC Kho ME Browman GP Burgers JS Cluzeau F Feder G . AGREE II: advancing guideline development, reporting and evaluation in health care. CMAJ. (2010) 182:E839–842. 10.1503/cmaj.090449 20603348 PMC3001530

[51] Bravo-Balado A Plata M Trujillo CG Caicedo JI Serrano A Ramos A . Is the development of clinical practice guidelines for non-neurogenic overactive bladder trustworthy? A critical appraisal using the Appraisal of Guidelines, Research and Evaluation (AGREE) II instrument. BJU Int. (2019) 123:921–2. 10.1111/bju.14684 30667143

[52] Radwan M Akbari Sari A Rashidian A Takian A Abou-Dagga S Elsous A. Appraising the methodological quality of the clinical practice guideline for diabetes mellitus using the AGREE II instrument: a methodological evaluation. JRSM Open. (2017) 8:2054270416682673. 10.1177/2054270416682673 28203385 PMC5298436

[53] Zhang J Xu J Zhang W Jiang M Liu J Xu L . Quality appraisal of guidelines on cancer-associated thrombosis using AGREE II instrument and analysis of current status of new oral anticoagulants. Clin Appl Thromb Hemost. (2019) 25:1076029619846562. 10.1177/1076029619846562 31025571 PMC6714899

[54] Kuznik A Bégo-Le-Bagousse G Eckert L Gadkari A Simpson E Graham CN . Economic evaluation of dupilumab for the treatment of moderate-to-severe atopic dermatitis in adults. Dermatol Ther. (2017) 7:493–505. 10.1007/s13555-017-0201-6 28933010 PMC5698200

[55] Wu KK Nguyen KB Sandhu JK Armstrong AW. Does location matter? Geographic variations in healthcare resource use for atopic dermatitis in the United States. J Dermatolog Treat. (2019) 2019:1–7. 10.1080/09546634.2019.1656796 31416361

